# Prospective study of physical activity and risk of postmenopausal breast cancer

**DOI:** 10.1186/bcr2190

**Published:** 2008-10-31

**Authors:** Michael F Leitzmann, Steven C Moore, Tricia M Peters, James V Lacey, Arthur Schatzkin, Catherine Schairer, Louise A Brinton, Demetrius Albanes

**Affiliations:** 1 Nutritional Epidemiology Branch, Division of Cancer Epidemiology and Genetics, National Cancer Institute, National Institutes of Health (NIH), Department of Health and Human Services (DHHS), 6120 Executive Blvd., MSC 7232, Bethesda, MD 20892, USA; 2 Institute of Epidemiology and Preventive Medicine, University Hospital Regensburg, Franz-Josef-Strauss-Allee 11, 93053 Germany; 3 Hormonal and Reproductive Epidemiology Branch, Division of Cancer Epidemiology and Genetics, National Cancer Institute, NIH, DHHS, 6120 Executive Blvd., MSC 7232, Bethesda, MD 20892, USA; 4 Biostatistics Branch, Division of Cancer Epidemiology and Genetics, National Cancer Institute, NIH, DHHS, 6120 Executive Blvd., MSC 7232, Bethesda, MD 20892, USA

## Abstract

**Introduction:**

To prospectively examine the relation of total, vigorous and non-vigorous physical activity to postmenopausal breast cancer risk.

**Methods:**

We studied 32,269 women enrolled in the Breast Cancer Detection Demonstration Project Follow-up Study. Usual physical activity (including household, occupational and leisure activities) throughout the previous year was assessed at baseline using a self-administered questionnaire. Postmenopausal breast cancer cases were identified through self-reports, death certificates and linkage to state cancer registries. A Cox proportional hazards regression was used to estimate the relative risk and 95% confidence intervals of postmenopausal breast cancer associated with physical activity.

**Results:**

During 269,792 person-years of follow-up from 1987 to 1998, 1506 new incident cases of postmenopausal breast cancer were ascertained. After adjusting for potential risk factors of breast cancer, a weak inverse association between total physical activity and postmenopausal breast cancer was suggested (relative risk comparing extreme quintiles = 0.87; 95% confidence interval = 0.74 to 1.02; p for trend = 0.21). That relation was almost entirely contributed by vigorous activity (relative risk comparing extreme categories = 0.87; 95% confidence interval = 0.74 to 1.02; p for trend = 0.08). The inverse association with vigorous activity was limited to women who were lean (ie, body mass index <25.0 kg/m^2^: relative risk = 0.68; 95% confidence interval = 0.54 to 0.85). In contrast, no association with vigorous activity was noted among women who were overweight or obese (ie, body mass index ≥ 25.0 kg/m^2^: relative risk = 1.18; 95% confidence interval = 0.93 to 1.49; p for interaction = 0.008). Non-vigorous activity showed no relation to breast cancer (relative risk comparing extreme quintiles = 1.02; 95% confidence interval = 0.87 to 1.19; p for trend = 0.86). The physical activity and breast cancer relation was not specific to a certain hormone receptor subtype.

**Conclusions:**

In this cohort of postmenopausal women, breast cancer risk reduction appeared to be limited to vigorous forms of activity; it was apparent among normal weight women but not overweight women, and the relation did not vary by hormone receptor status. Our findings suggest that physical activity acts through underlying biological mechanisms that are independent of body weight control.

## Introduction

Breast cancer is the most common malignancy among women in the US. It was estimated that 178,000 new breast cancer cases would occur in 2007 and that 40,000 women would die from the disease [1]. Two recent systematic reviews [2,3] concluded that data from epidemiological studies strongly suggest an inverse relation of physical activity to breast cancer, but that results are limited by an inadequate characterisation of the influences of type, frequency, duration and intensity of activity. The reviews called for further research aimed at elucidating what aspects of physical activity contribute most towards decreasing risk. We therefore examined total, vigorous and non-vigorous activity of all types (household, occupational and recreational activities) in relation to breast cancer risk in a large prospective cohort of women enrolled in the Breast Cancer Detection Demonstration Project (BCDDP) Follow-up Study.

## Materials and methods

### Study population

The BCDDP was a mammography demonstration program jointly sponsored by the National Cancer Institute and the American Cancer Society [4]. The program enrolled 283,222 participants who underwent breast examinations between 1973 and 1980 in one of 29 screening centres located in 27 cities across the US. In 1979, the National Cancer Institute established the BCDDP Follow-up Study of 64,182 women enrolled in the original BCDDP screening study. The BCDDP Follow-up Study cohort included: all women diagnosed with breast cancer during BCDDP screening (n = 4275); all women with benign breast disease confirmed by biopsy but with no malignant disease (n = 25,114); all women who were recommended by the BCDDP for surgical consultation but for whom no procedure was performed (n = 9628); and a random sample of BCDDP participants who had no surgery or recommendation for surgical consultation during screening (n = 25,165).

Since its inception in 1979, the BCDDP Follow-up Study has proceeded in several phases. Phase 1 from 1979 to 1986 involved a telephone interview at baseline and up to six annual telephone follow-up interviews through 1986. Phases 2, 3 and 4 each used individual, self-administered questionnaires that were mailed between 1987 and 1989, 1993 and 1995, and 1995 and 1998, respectively, to all cohort members who were presumed alive at the end of the previous phase. The questionnaires were designed to gather basic demographic information, to update exposures to various potential risk factors for chronic disease and to ascertain newly diagnosed cancer. Non-respondents to mailed questionnaires were interviewed by telephone, if possible. The BCDDP Follow-up Study was approved by the Institutional Review Board of the National Cancer Institute, and informed consent was obtained from all participants.

### Population for analysis

The phase 2 questionnaire, which first requested information on physical activity and formed the baseline of the current report, was returned by 81% of the original 64,182 participants of the BCDDP Follow-up Study. Of these 51,696 women who were aged 40 to 93 years at baseline, we excluded from the current analysis those with a breast cancer diagnosis before phase 2 (n = 5152), women with other previous cancers other than non-melanoma skin cancer (n = 2877), those who were premenopausal throughout the duration of follow-up (n = 102), those with missing or inadequate information on physical activity (n = 10,314), and those who provided inadequate data on calorie intake (n = 658), body weight (n = 300) or height during any phase (n = 26). The primary analyses in the current study are based on the remaining 32,269 women, who were followed from 1987 to 1998. Of these, 91% completed the phase 3 questionnaire, 85% completed the phase 4 questionnaire and 94% completed either the phase 3 or phase 4 questionnaires.

Most participants in the population for analysis were Caucasian (89%), with small percentages being African American (3%), Asian (5%), Hispanic (2%) or of other or missing race/ethnicity (1%). The majority of participants (85%) were postmenopausal at baseline.

### Assessment of physical activity

Usual physical activity during the previous year was assessed by asking subjects to estimate the number of hours per typical weekday and weekend day they spent engaging in moderate and vigorous physical activities. The questionnaire encompassed an extensive list of examples of moderate and vigorous activities that covered household, occupational, and recreational or sporting activities. Examples of moderate activity (referred to as non-vigorous activity) included light housework, vacuuming, washing clothes, painting, home repairs, lawn mowing, general gardening, raking, light sports or exercise, walking, hiking, light jogging, recreational tennis, bowling, golf and bicycling on a level ground. Examples of vigorous activity included heavy housework such as scrubbing floors or washing windows, heavy yard-work, digging in the garden, chopping wood, strenuous sports or exercise, running, fast jogging, competitive tennis, aerobics, bicycling on hills and fast dancing. Our questionnaire also queried about the time spent sleeping and engaging in sedentary behaviours (examples included sitting, office work, driving a car, occupations that involved standing or walking, watching television, and reading). We did not consider the information regarding sleeping and sedentary behaviours in our current analysis of physical activity.

The reported hours per day spent in non-vigorous and vigorous activities were converted to weekly average hours using the following formula: [(weekday hours × 5) + (weekend hours × 2)]/7. A weekly physical activity score was calculated for non-vigorous and vigorous activity by multiplying the time spent at each category of activity by its energy expenditure requirements, expressed as metabolic equivalent tasks (METs) [5]. One MET is defined as the energy expended sitting quietly, which is equivalent to an oxygen uptake of 3.5 ml/kg/minute for an adult weighing 70 kg [6]. Because categories included numerous individual activities, they were assigned an average MET value: four METs for non-vigorous activity and six METs for vigorous activity. A total weekly MET-hour score (total physical activity) was calculated by summing up the weekly expenditures from non-vigorous and vigorous activities. The methods used to calculate the physical activity variables build on previous work in this cohort [7].

Our method of assessing physical activity has not undergone a direct comparison with physical activity logs or other validation tools. However, our physical activity assessment is similar to that employed in the Framingham Heart Study, which demonstrated a correlation between questionnaire-based and indirect calorimetry-based physical activity of 0.43 [8]. In addition, our physical activity measure includes selected components of the College Alumnus Physical Activity Questionnaire [9], an assessment tool that is positively correlated with maximum oxygen uptake, percentage body fat, high-density lipoprotein levels and body mass index (BMI) [10,11].

### Identification of breast cancer cases

We identified new incident breast cancer cases through pathology reports, linkage to state cancer registries, the National Death Index (from 31 December 1997) and from self-reports. Pathology reports were sought for all self-reported cancers. In addition, about 80% of the cohort was linked to state cancer registries using the state of last known residence at the time of the phase 4 questionnaire mailing. Using these methods, we ascertained a total of 1506 postmenopausal breast cancer cases, of which 260 (17%) were classified as *in situ *cancers. Of the breast cancer cases, 1147 (76%) were identified based on information collected from pathology reports, 198 (13%) were ascertained using information provided by state cancer registries and 161 (11%) were self-reported breast cancers with no pathology report or data from a state registry. We had an insufficient number of premenopausal breast cancer cases to provide stable individual risk estimates; thus, we limited all analyses to postmenopausal breast cancer. Sensitivity analyses were conducted excluding the 260 cases with *in situ *breast cancer and excluding the 161 cases who self-reported breast cancer, but for whom we lacked confirmation. Results were similar to the overall result and are not reported separately.

We also evaluated breast cancer risk according to hormone receptor status, information of which was obtained from cancer registries serving our cohort. The threshold for a positive hormone receptor status was 10 fmol or more of receptor per milligram of total protein. Because a proportion of our cases was identified through self-reporting and because certain cancer registries do not routinely collect data regarding hormone receptor status, information on hormone receptor status was unavailable for 639 breast cancer cases (42%). Thus, analyses regarding hormone receptor status disregarded those cases. Women for whom hormone receptor data was available did not differ by age, education level or physical activity from women for whom such information was not available (data not shown).

### Statistical analysis

Person-time of follow-up for each participant began at the return date of the phase 2 questionnaire and it ended at the date of breast cancer diagnosis, death from any cause or date of return of the phase 4 questionnaire. The exit date of a participant who was lost to follow-up was assigned as the date of last contact during 1995 and 1998 or, if the individual could not be contacted, the date of their last completed questionnaire plus the mean time between completion of successfully completed questionnaires derived from the cohort as a whole.

We used Cox proportional hazards regression [12] to estimate the relative risk (RR) of breast cancer using person-years of follow-up as a metric time and adjusting for age at baseline (continuous), family history of breast cancer (any first- or second-degree relative; yes, no or unknown), history of benign breast disease (any benign breast disease before study baseline, including during the screening stages; yes or no), BCDDP screening subgroup (women who had undergone a breast biopsy as part of the BCDDP screening but lacked evidence of malignant disease, women who were recommended for surgical consultation as part of the BCDDP but for whom the procedure was not performed, and women who represented a random sample of BCDDP participants who had no surgery or recommendation for surgical consultation during BCDDP screening), height (continuous), age at menarche (11 and younger, 12, 13 or 14 and older years of age), age at menopause, including women whose periods stopped as a result of hysterectomy (age at which menstrual period stopped for at least three continuous months; younger than 47 or 47 years of age or older), age at first live birth (continuous), history of oral contraceptive use (yes or no), menopausal hormone therapy (ever or never), education attainment (less than or equal to high school or greater than high school), cigarette smoking (ever or never), and intakes of energy-adjusted dietary fat (continuous) and alcohol (continuous). Terms for total and non-vigorous physical activity were divided into quintiles. Vigorous activity was grouped such that women who reported a daily average of zero hours were placed into one category and those who reported nonzero values were divided into quartiles.

Information on education and height was collected on entry into the original screening program. Data regarding age at menarche, age at first live birth and oral contraceptive use was collected during phase 1 of the study. Current body weight, diet, smoking habits, information on menopause including hysterectomy status, and menopausal hormone therapy use was assessed using the phase 2 questionnaire.

We conducted secondary analyses that were additionally adjusted for mammography subsequent to the screening phase (yes or no), history of bone fracture after age 45 years (yes or no) and history of osteoporosis (yes or no) to assess the potential for confounding by those variables. Because BMI potentially represents a causal pathway linking increased physical activity to decreased breast cancer risk, we did not include BMI in our primary analyses. However, additional models contained BMI to assess the impact of physical activity on breast cancer independent of its effect on body weight. In addition, the relation of physical activity to breast cancer risk was evaluated within strata of BMI. We used a BMI cut-off point of 25.0 kg/m^2 ^to distinguish lean or normal weight women from overweight or obese women.

Statistical interaction of BMI and other known or suspected breast cancer risk factors was examined by entering the cross-product term for physical activity and a given variable with the main effects terms for each in the appropriate multivariate model, the coefficients for which were evaluated using a Wald test. Tests for linear trend were conducted by entering a single ordinal variable corresponding to the median of the physical activity category into the model. All hypothesis tests were two-sided and associations were considered to be statistically significant if p < 0.05. All analyses were conducted using SAS release 9.1.3 (SAS Institute, Cary, NC).

## Results

In our study population, the range of total physical activity varied about 2.5-fold between the means of extreme quintiles. Women reported spending an average of 1.2 hours a day in vigorous activity and 5.9 hours a day in non-vigorous activity. We first examined physical activity in relation to potential confounding factors for breast cancer (Table 1). Women who were physically more active tended to be slightly leaner, to have never smoked, to have a lower level of education and to have greater parity than physically less active women. Differences between physically active and physically inactive women with respect to age at first birth, age at menopause, history of oral contraceptive use and menopausal hormone therapy tended to be minimal.

**Table 1:** Baseline characteristics according to quintiles of total physical activity in 32,269 women in the Breast Cancer Detection Demonstration Project (BCDDP)*

**Characteristic**	**Quintile of total physical activity**				
	1	2	3	4	5
	
Participants (n)	6453	6454	6441	6472	6449
Age (years)	61.7	61.1	61.3	60.8	61.0
MET-hours/week (range) †	105 to 244	245 to 297	298 to 339	340 to 394	395 to 721
Vigorous physical activity (hours/week)	0.9	3.6	5.4	10.3	22.7
Non-vigorous physical activity (hours/week)	14.8	32.4	46.5	54.5	59.7
Body mass index (kg/m^2^)	25.4	25.0	24.9	24.7	24.6
Height (inches)	64.3	64.2	64.2	64.2	64.1
Ever smoked cigarettes (%)	46.9	44.6	43.4	42.1	40.7
Education (% more than high school)	55.0	50.0	46.8	46.1	42.0
Age at first birth (years) ‡	24.0	23.9	23.8	23.9	23.5
Parity (number of births) ‡	2.8	2.8	2.9	2.9	3.0
Age at menarche (years)	12.7	12.7	12.8	12.8	12.9
Age at menopause (years)	50.4	50.3	50.2	50.4	50.3
History of oral contraceptive use (%)	33.8	33.2	33.4	32.8	31.1
Current menopausal hormone therapy (%)	55.3	54.7	55.8	55.7	54.1
Family history of breast cancer (%) ¶	34.0	35.1	34.1	34.4	33.6
History of benign breast disease (%) §	55.5	56.0	55.4	55.6	55.2
Total energy intake (kcal/day)	1252	1256	1266	1271	1277
Dietary fat intake (g/day) ||	46.9	46.5	46.3	46.0	45.9
Alcohol intake (g/day)	4.5	4.4	4.1	4.0	3.6

* All values (except age and range of MET-hours/week) are directly standardised to the age distribution of the cohort at study baseline.

† MET = metabolic equivalent task. The MET value is the caloric need per kilogram of body weight per hour of activity divided by the caloric need per kilogram of body weight per hour at rest.

‡ Among parous women only.

¶Family history of breast cancer includes first- and second-degree relatives with a history breast cancer.

§The BCDDP Follow-up Study includes a disproportionate number of women with benign breast disease due to the design of the original BCDDP.

||Adjusted for total energy intake.

During 269,792 person-years of follow-up, we documented 1506 cases of postmenopausal breast cancer. In age-adjusted analyses, total physical activity was inversely related to risk of postmenopausal breast cancer (Table 2). The most active women had an age-adjusted RR of 0.81 (95% confidence interval (CI) = 0.69 to 0.95; p = 0.03) as compared with the least active women. The relative risk was slightly attenuated and became statistically non-significant after additional adjustment for potential confounding variables (RR = 0.86; 95% CI = 0.73 to 1.01; p = 0.15). Additional adjustment for BMI did not change the relationship (RR = 0.87; 95% CI = 0.74 to 1.02; p = 0.21). Results were virtually identical in secondary models that were also adjusted for mammography subsequent to the screening phase, history of bone fracture after age 45 years, and history of osteoporosis (0.87; 95% CI = 0.74 to 1.02; p = 0.22).

**Table 2:** Relative risk of postmenopausal breast cancer in relation to total physical activity among US women in the Breast Cancer Detection Demonstration Project (BCDDP; 1987 to 1998)*

**Variable**	**Quintile of total physical activity**					**p**
	1	2	3	4	5	
		
**Physical activity**						
MET-hours/week (range)	105 to 244	245 to 297	298 to 339	340 to 394	395 to 721	
Cases	320	300	308	312	266	
Person-years	52,858	53,967	54,019	54,487	54,461	
Age-adjusted RR (95% CI)	1.0	0.92 (0.79 to 1.08)	0.94 (0.81 to 1.10)	0.95 (0.82 to 1.12)	0.81 (0.69 to 0.95)	0.03
Multivariate RR † (95% CI)	1.0	0.92 (0.79 to 1.08)	0.97 (0.83 to 1.14)	0.98 (0.84 to 1.15)	0.86 (0.73 to 1.01)	0.15
Multivariate RR ‡ (95% CI)	1.0	0.93 (0.80 to 1.09)	0.98 (0.84 to 1.14)	1.00 (0.85 to 1.16)	0.87 (0.74 to 1.02)	0.21

* CI = confidence interval; MET = metabolic equivalent task; RR = relative risk.

† The multivariate model included the following: age at baseline (continuous), family history of breast cancer (any first- or second-degree relative; yes, no or unknown), history of benign breast disease (any benign breast disease prior to study baseline, including during the screening stages; yes or, no), breast cancer screening history (women who had undergone a breast biopsy as part of the BCDDP screening but lacked evidence of malignant disease, women who were recommended for surgical consultation as part of the BCDDP but for whom the procedure was not performed, or women who represented a random sample of BCDDP participants who had no surgery or recommendation for surgical consultation during BCDDP screening), height (continuous), age at menarche (≤ 11, 12, 13 or 14+ years of age), age at menopause (age at which menstrual period stopped for at least three continuous months; <47 or 47+ years of age), age at first live birth (continuous), history of oral contraceptive use (yes or no), menopausal hormone therapy (ever or never), education attainment (less than or equal to high school, or greater than high school), cigarette smoking (ever or never), and intakes of energy-adjusted dietary fat (continuous) and alcohol (continuous).

‡ Also controlled for body mass index (continuous).

The apparent inverse association between total physical activity and postmenopausal breast cancer was virtually entirely contributed by vigorous physical activity (Table 3). After adjusting for multiple confounding factors including non-vigorous activity and BMI, women in the highest category of vigorous activity had an RR of 0.87 (95% CI = 0.74 to 1.02; p = 0.08) compared with women with no vigorous activity. When we examined deciles of vigorous physical activity, the RR in the top category (n = 70) was 0.79 (95% CI = 0.61 to 1.01) (data not tabulated). In contrast, non-vigorous activity showed no association with risk for postmenopausal breast cancer (multivariate RR comparing extreme quintiles = 1.02; 95% CI = 0.87 to 1.19; p = 0.86).

**Table 3:** Relative risk of postmenopausal breast cancer in relation to vigorous and non-vigorous physical activity among US women in the Breast Cancer Detection Demonstration Project (BCDDP: 1987 to 1998)*

**Variable**	**Category of vigorous or non-vigorous physical activity**					**p**
	1	2	3	4	5	
		
**Vigorous physical activity**						
MET-hours/week	0	0.1 to 48.9	49.0 to 70.0	70.1 to 126.0	126.1 to 588.0	
Cases	614	236	234	218	204	
Person-years	101,276	40,772	44,949	41,072	41,772	
Age-adjusted RR (95% CI)	1.0	1.00 (0.86 to 1.17)	0.89 (0.76 to 1.03)	0.90 (0.77 to 1.06)	0.82 (0.70 to 0.96)	0.008
Multivariate RR † §(95% CI)	1.0	0.98 (0.84 to 1.14)	0.88 (0.76 to 1.03)	0.91 (0.78 to 1.07)	0.86 (0.73 to 1.01)	0.06
Multivariate RR ‡ §(95% CI)	1.0	0.98 (0.85 to 1.15)	0.89 (0.76 to 1.04)	0.92 (0.79 to 1.08)	0.87 (0.74 to 1.02)	0.08
**Non-vigorous physical activity**						
MET hours per week	0 to 84.0	84.1 to 140.0	140.1 to 188.0	188.1 to 229.0	229.1 to 504.0	
Cases	317	314	283	287	305	
Person-years	53,660	56,791	52,224	53,001	54,116	
Age-adjusted RR (95% CI)	1.0	0.94 (0.80 to 1.09)	0.91 (0.78 to 1.07)	0.91 (0.77 to 1.06)	0.95 (0.81 to 1.11)	0.48
Multivariate RR † || (95% CI)	1.0	0.99 (0.84 to 1.16)	0.98 (0.83 to 1.16)	0.97 (0.82 to 1.14)	1.01 (0.86 to 1.18)	0.96
Multivariate RR ‡ || (95% CI)	1.0	0.99 (0.84 to 1.16)	0.99 (0.84 to 1.16)	0.97 (0.82 to 1.14)	1.02 (0.87 to 1.19)	0.86

* CI = confidence interval; MET = metabolic equivalent task; RR = relative risk.

† The multivariate model included the following: age at baseline (continuous), family history of breast cancer (any first- or second-degree relative; yes, no or unknown), history of benign breast disease (any benign breast disease prior to study baseline, including during the screening stages; yes or no), breast cancer screening history (women who had undergone a breast biopsy as part of the BCDDP screening but lacked evidence of malignant disease, women who were recommended for surgical consultation as part of the BCDDP but for whom the procedure was not performed, or women who represented a random sample of BCDDP participants who had no surgery or recommendation for surgical consultation during BCDDP screening), height (continuous), age at menarche (≤ 11, 12, 13 or 14+ years of age), age at menopause (age at which menstrual period stopped for at least three continuous months; <47 or 47+ years of age), age at first live birth (continuous), history of oral contraceptive use (yes or no), menopausal hormone therapy (ever or never), education attainment (less than or equal to high school or greater than high school), cigarette smoking (ever or never), and intakes of energy-adjusted dietary fat (continuous) and alcohol (continuous).

‡ Also controlled for body mass index (continuous).

§Also controlled for non-vigorous physical activity (quintiles).

||Also controlled for vigorous activity (5 categories).

We investigated the relation of physical activity to postmenopausal breast cancer within strata of BMI (Table 4). Among normal weight and lean women (BMI < 25.0 kg/m^2^), the multivariate RRs comparing extreme categories of total and vigorous activity were 0.76 (95% CI = 0.61 to 0.94; p = 0.03) and 0.68 (95% CI = 0.54 to 0.85; p = 0.002), respectively. The test for interaction between vigorous activity and BMI was statistically significant (p = 0.008). In contrast, the relations of total and non-vigorous activity to breast cancer were not statistically significantly modified by BMI (p = 0.19 and 0.47, respectively).

**Table 4:** Relative risk of postmenopausal breast cancer in relation to total, vigorous and non-vigorous physical activity in subgroups defined by body mass index among US women in the Breast Cancer Detection Demonstration Project (BCDDP; 1987 to 1998)*

**Variable**	**No. of cases**	**Category of physical activity**					**P-value, test for trend**	**P-value, test for interaction**
		**1**	**2**	**3**	**4**	**5**		
				
		*Total physical activity*						
**Body mass index**								
< 25.0 kg/m^2^	876	1.0	0.88 (0.71 to 1.08)	0.92 (0.75 to 1.12)	0.91 (0.74 to 1.11)	0.76 (0.61 to 0.94)	0.03	
≥ 25.0 kg/m^2^	630	1.0	1.01 (0.79 to 1.29)	1.07 (0.84 to 1.37)	1.12 (0.88 to 1.43)	1.06 (0.82 to 1.36)	0.48	0.19
		*Vigorous physical activity*						
**Body mass index**								
< 25.0 kg/m^2^	876	1.0	0.87 (0.72 to 1.07)	0.81 (0.67 to 0.99)	0.90 (0.73 to 1.10)	0.68 (0.54 to 0.85)	0.002	
≥ 25.0 kg/m^2^	630	1.0	1.15 (0.91 to 1.46)	1.00 (0.79 to 1.27)	0.93 (0.72 to 1.21)	1.18 (0.93 to 1.49)	0.33	0.008
		*Non-vigorous physical activity*						
**Body mass index**								
< 25.0 kg/m^2^	876	1.0	0.97 (0.79 to 1.20)	1.05 (0.85 to 1.31)	0.97 (0.78 to 1.20)	0.98 (0.79 to 1.21)	0.85	
≥ 25.0 kg/m^2^	630	1.0	1.01 (0.79 to 1.28)	0.90 (0.69 to 1.16)	0.98 (0.77 to 1.27)	1.09 (0.85 to 1.39)	0.59	0.47

CI = confidence interval; RR = relative risk. The multivariate model included the following: age at baseline (continuous), family history of breast cancer (any first- or second-degree relative; yes, no or unknown), history of benign breast disease (any benign breast disease prior to study baseline, including during the screening stages; yes or no), breast cancer screening history (women who had undergone a breast biopsy as part of the BCDDP screening but lacked evidence of malignant disease, women who were recommended for surgical consultation as part of the BCDDP but for whom the procedure was not performed, or women who represented a random sample of BCDDP participants who had no surgery or recommendation for surgical consultation during BCDDP screening), height (continuous), age at menarche (≤ 11, 12, 13 or 14+ years of age), age at menopause (age at which menstrual period stopped for at least three continuous months; <47 or 47+ years of age), age at first live birth (continuous), history of oral contraceptive use (yes or no), menopausal hormone therapy (ever or never), education attainment (less than or equal to high school or greater than high school), cigarette smoking (ever or never), and intakes of energy-adjusted dietary fat (continuous) and alcohol (continuous). Within each stratum, the group with lowest vigorous physical activity served as the reference group.

† Body mass index is the weight in kilograms divided by height in metres squared.

We subdivided breast cancer cases according to hormone receptor status and found no evidence that the relation between physical activity and breast cancer was specific to a certain hormone receptor subtype, neither before (data not shown) or after adjustment for BMI (Table 5).

**Table 5:** Relative risk of oestrogen receptor positive (ER+) breast cancer and oestrogen receptor negative (ER-) breast cancer in relation to vigorous physical activity among US women in the Breast Cancer Detection Demonstration Project (BCDDP; 1987 to 1998)*

**Variable**	**No. of Cases**	**Category of vigorous physical activity**					**p**
		**1**	**2**	**3**	**4**	**5**	
			
**ER/PR receptor status**							
ER+	706	1.0	0.94	0.93	0.93	0.91 (0.72 to 1.15)	0.42
PR+	588	1.0	1.13	0.97	1.03	0.93 (0.72 to 1.22)	0.54
ER+/PR+	555	1.0	1.08	0.97	1.02	0.93 (0.71 to 1.23)	0.61
ER+/PR-	115	1.0	2.40	0.89	0.97	0.49 (0.10 to 2.32)	0.22
ER-	161	1.0	1.36	0.79	1.02	0.74 (0.44 to 1.27)	0.22
PR-	248	1.0	0.77	0.74	0.80	0.77 (0.52 to 1.15)	0.23
ER-/PR+	29	1.0	0.55	0.60	0.56	0.81 (0.46 to 1.41)	0.43
ER-/PR-	126	1.0	1.02	0.80	1.06	0.76 (0.42 to 1.35)	0.43
**Combination of ER receptor status and BMI**							
ER+, BMI < 25.0 kg/m^2^	417	1.0	0.79	0.87	0.95	0.75 (0.54 to 1.03)	0.15
ER+, BMI ≥ 25.0 kg/m^2^	289	1.0	1.20	1.01	0.86	1.19 (0.83 to 1.69)	0.60
**Combination of ER/PR receptor status and BMI**							
ER+/PR+, BMI < 25.0 kg/m^2^	327	1.0	0.94	0.93	1.09	0.81 (0.56 to 1.17)	0.43
ER+/PR+, BMI ≥ 25.0 kg/m^2^	228	1.0	1.29	1.02	0.89	1.14 (0.76 to 1.71)	0.81

BMI = body mass index; CI = confidence interval; ER = oestrogen receptor; PR = progesterone receptor; RR = relative risk. The multivariate model included the following: age at baseline (continuous), family history of breast cancer (any first or second degree relative; yes, no or unknown), history of benign breast disease (any benign breast disease prior to study baseline, including during the screening stages; yes or no), breast cancer screening history (women who had undergone a breast biopsy as part of the BCDDP screening but lacked evidence of malignant disease, women who were recommended for surgical consultation as part of the BCDDP but for whom the procedure was not performed, or women who represented a random sample of BCDDP participants who had no surgery or recommendation for surgical consultation during BCDDP screening), height (continuous), age at menarche (≤ 11, 12, 13 or 14+ years of age), age at menopause (age at which menstrual period stopped for at least three continuous months; <47 or 47+ years of age), age at first live birth (continuous), history of oral contraceptive use (yes or no), menopausal hormone therapy (ever or never), education attainment (less than or equal to high school or greater than high school), cigarette smoking (ever or never), and intakes of energy-adjusted dietary fat (continuous) and alcohol (continuous). Within each stratum, the group with lowest vigorous physical activity served as the reference group.

The relations of total, vigorous and non-vigorous activity with postmenopausal breast cancer risk were not further modified by other potential breast cancer risk factors, including age, breast cancer screening history, family history of breast cancer, history of benign breast disease, age at menarche, age at first birth, parity, menopausal hormone use, age at menopause, height, race, education attainment, cigarette smoking, and dietary intakes of alcohol and total dietary fat (p > 0.05).

## Discussion

We found that a higher amount of vigorous physical activity was associated with a small, statistically non-significant decrease in postmenopausal breast cancer risk in our cohort as a whole. However, when we evaluated the relation of vigorous activity to breast cancer among women who were of normal weight, the association became markedly stronger, with risk among women reporting the highest amount of vigorous activity decreasing by about 30% compared with women with no vigorous activity. In contrast, no association between vigorous activity and breast cancer was noted among women who were overweight or obese. In addition, we observed no association between non-vigorous activity and breast cancer, neither in the cohort as a whole or after stratification by BMI. We also found no heterogeneity of the physical activity and breast cancer association according to hormone receptor status.

As published in a recent meta-analysis of the available literature [2], most previous studies that examined physical activity in relation to postmenopausal breast cancer reported risk reductions of 20% to 80% for the highest compared with the lowest physical activity levels. Some [13-15] but not all previous investigations [16-21] noted a significant inverse relation with vigorous activity but detected a weaker or no association with non-vigorous activity. That vigorous activity may afford greater apparent protection from breast cancer development than non-vigorous activity is supported by findings that greater intensity of physical activity or level of physical training is related to more pronounced perturbations of sex hormone levels and menstrual function [22]. However, data from randomised trials among postmenopausal women demonstrate that circulating levels of androstenedione and oestrone, important modulators of breast cancer risk, are lowest among women assigned to engaging in the most amount of overall activity [23-25], suggesting that the amount of activity is more relevant than the intensity of activity, at least for reducing oestrogen levels.

We found that the relation of vigorous activity to breast cancer was modified by BMI, with an inverse physical activity association limited to lean or normal weight women. This is consistent with numerous previous studies [16,17,26-35] observing an apparent protective effect of physical activity on breast cancer risk that was more evident among lean women than overweight women. However, data regarding this issue are equivocal, with some reports noting a more pronounced inverse physical activity and breast cancer relation among overweight women compared with lean women [36-38], and other investigations finding no heterogeneity of the association between physical activity and breast cancer according to adiposity level [14,19,39-46].

That increased physical activity was related to a more pronounced reduction in risk for breast cancer among lean women compared with overweight women and the observation that the influence of physical activity was almost unaffected by adjustment for BMI suggests an underlying biological mechanism that is independent of body weight control. Possible mechanisms through which physical activity may protect against breast cancer that are independent of BMI include reduced exposure to growth factors, enhanced immune function and decreased chronic inflammation, variables that are related both to greater physical activity and to lower breast cancer risk [47].

A non-causal explanation for a stronger inverse relation of vigorous activity among lean women compared with overweight women is that heavier women may not exercise as intensely as lean women. Moreover, non-vigorous activities performed by overweight women, such as light housework, general gardening and light sports, may be misreported as vigorous activities among overweight individuals.

We found very little evidence that the relation of physical activity to breast cancer varied according to hormone receptor subtype, although information on hormone receptor status was available for only a portion of the study subjects. This finding is in line with several previous studies on this topic [14,26,48,49]. Some investigations did find heterogeneity of the physical activity relation by breast cancer hormone receptor status. For example, in the California Teachers Study [15] increased levels of both strenuous and moderate recreational activity were related to decreased risks of oestrogen receptor (ER)-negative, but not ER-positive breast cancers. In the Iowa Women's Study [50] enhanced physical activity level was associated with decreased risks of ER-positive/progesterone receptor (PR)-positive, ER-positive/PR-negative and ER-negative/PR-negative breast cancers. However, the only breast cancers for which a statistically significant inverse relation remained after adjustment for BMI were ER-positive/PR-negative types. A case-control study found a suggestive inverse relation of current recreational activity with ER-negative breast cancers and an apparent increased risk with ER-positive breast cancers [51]. However, the directionality of those associations was reversed when considering adolescent recreational activity [51]. Taken together, available data concerning the relation of physical activity to breast cancer according to hormone receptor subtype are currently not sufficiently consistent to draw firm conclusions.

Despite reasonable consistency of our findings with those from the existing literature [2], a direct comparison of activity levels among women in our study with those from previous investigations is difficult because of substantial variation in the design of physical activity questionnaires across available breast cancer studies. Our questionnaire format may have been associated with some degree of over-reporting of activity as suggested by circumstantial data showing that self-administered activity questions can lead to inflated estimates of the reported time spent engaging in physical activity as compared with interviewer-administered assessments [52]. However, the main possible correlates of activity over-reporting, including age and body size, were accounted for in our multivariate statistical analyses.

Our physical activity tool included an assessment of physical activity intensity, which appeared to be sufficiently comprehensive to distinguish between vigorous and non-vigorous forms of activity. Nonetheless, we cannot rule out the possibility of misclassification of non-vigorous and vigorous types of activities in our study, which would tend to attenuate relative risk estimates due to random error in capturing and quantifying the most relevant intensity of physical activity. Although our questionnaire was aimed at assessing all types of physical activity, it lacked the level of refinement necessary to evaluate specific individual activities, such as walking.

Women who were more physically active in our cohort tended to have a lower education level than those who were less active. Thus, we cannot entirely rule out the possibility that the inverse association between vigorous activity and breast cancer observed in our study was partly explained by lower socioeconomic status related both to increased activity levels and to decreased risk of breast cancer. On the other hand, lower socioeconomic status among physically active women indicates that a combination of household and occupational activities represented a quantitatively greater contribution to total physical activity among women in our cohort than in other studies in which physical activity assessments were limited to recreational or sports activities, which tend to track positively with elements of a healthy lifestyle and also bear the potential for confounding [51].

Our analysis was based on a single, baseline assessment of physical activity, which may not have precisely represented long-term activity habits. Data on physical activity throughout the life course would have been important in examining early-age or long-term physical activity patterns in relation to subsequent breast cancer risk [49]. Our findings suggest that physical activity in mid to late adulthood is aetiologically relevant for influencing breast cancer risk.

We did not encompass possible alterations in activity levels during follow-up. Conceivably, our single point-in-time measure of physical activity misclassified women with respect to their habitual activity level during the study. In particular, women in our cohort who had recently undergone surgical consultation or therapy for benign breast disease may have subsequently modified their physical activity habits. Because our physical activity questionnaire was designed to assess recent exposure to physical activity, any impact of breast cancer screening on activity levels during the subsequent period of follow-up would have diminished the ability of our instrument to reflect true long-term physical activity levels. However, because our physical activity assessment preceded the diagnosis of breast cancer, potential misclassification of physical activity would have tended to be non-differential with respect to disease status, which would have resulted in an attenuation of the true association. In addition, findings from other prospective cohorts [53] indicate that physical activity levels tend to track well over time, which helps explain why investigations employing a single baseline measure have uncovered relevant associations between physical activity and breast cancer using that strategy.

We also were not able to encompass changes in potential confounding variables or effect modifiers during follow-up. Large changes in levels of variables such as BMI or menopausal hormone therapy use could have influenced our findings.

Notable strengths of our study include its large sample size, prospective design, high follow-up rate, and availability of relevant known or suspected breast cancer risk factors. These features enabled us to minimise major bias and confounding. Consistent with the majority of previous investigations of physical activity and breast cancer [2], few variables confounded our findings.

An important limitation of our study is our reliance on self-reported physical activity, a method that is prone to both systematic and random errors. A further potential limitation is that our cohort comprised predominantly Caucasian women who volunteered to participate in a long-term follow-up study. Thus, our findings may not be strictly relevant to all women. In addition, women who had undergone a previous breast biopsy were over-sampled in our study, potentially further decreasing the general nature of our results. However, the relation of physical activity to breast cancer was not modified by breast cancer screening history in our study, suggesting broad applicability of our results.

## Conclusions

In conclusion, our results support the hypothesis of an inverse association between physical activity and postmenopausal breast cancer. Risk reduction appeared to be limited to vigorous forms of activity. Our data also suggest that the potential protective effect of vigorous activity on postmenopausal breast cancer risk is most apparent among lean or normal weight rather than overweight women. Possible reasons for such heterogeneity include both causal and non-causal mechanisms. The physical activity and breast cancer relation did not vary according to hormone receptor status. Thus, that particular aspect of our study revealed little additional mechanistic insight into breast cancer aetiology. Future studies designed to evaluate in detail the relations of individual components of physical activity, including specific vigorous and non-vigorous activities throughout the life course in relation to risk of breast cancer overall and by hormone receptor phenotype will allow further insights into possible biological mechanisms of breast carcinogenesis.

## Abbreviations

BCDDP: Breast Cancer Detection Demonstration Project; BMI: body mass index; CI: confidence interval; ER: oestrogen receptor; MET: metabolic equivalent task; PR: progesterone receptor; RR: relative risk.

## Competing interests

The authors declare that they have no competing interests.

## Authors' contributions

MFL and SCM carried out the statistical analysis. MFL drafted the manuscript. MFL, SCM, TMP, JVL, AS, CS, LAB and DA participated in the design and co-ordination of the study. MFL and DA conceived of the study. All authors read and approved the final manuscript.

## References

[B1] Jemal A, Siegel R, Ward E, Murray T, Xu J, Thun MJ (2007). Cancer statistics, 2007. CA Cancer J Clin.

[B2] Monninkhof EM, Elias SG, Vlems FA, Tweel I van der, Schuit AJ, Voskuil DW, van Leeuwen FE (2007). Physical activity and breast cancer: a systematic review. Epidemiology.

[B3] Friedenreich CM, Cust AE (2008). Physical activity and breast cancer risk: impact of timing, type and dose of activity and population subgroup effects. Br J Sports Med.

[B4] Morrison AS, Brisson J, Khalid N (1988). Breast cancer incidence and mortality in the breast cancer detection demonstration project. J Natl Cancer Inst.

[B5] Ainsworth BE, Haskell WL, Whitt MC, Irwin ML, Swartz AM, Strath SJ, O'Brien WL, Bassett DR, Schmitz KH, Emplaincourt PO, Jacobs DR, Leon AS (2000). Compendium of physical activities: an update of activity codes and MET intensities. Med Sci Sports Exerc.

[B6] Ainsworth BE, Haskell WL, Leon AS, Jacobs DR, Montoye HJ, Sallis JF, Paffenbarger RS (1993). Compendium of physical activities: classification of energy costs of human physical activities. Med Sci Sports Exerc.

[B7] Colbert LH, Lacey JV, Schairer C, Albert P, Schatzkin A, Albanes D (2003). Physical activity and risk of endometrial cancer in a prospective cohort study (United States). Cancer Causes Control.

[B8] Kannel WB, Sorlie P (1979). Some health benefits of physical activity. The Framingham Study. Arch Intern Med.

[B9] Paffenbarger RS, Wing AL, Hyde RT (1978). Physical activity as an index of heart attack risk in college alumni. Am J Epidemiol.

[B10] Ainsworth BE, Leon AS, Richardson MT, Jacobs DR, Paffenbarger RS (1993). Accuracy of the College Alumnus Physical Activity Questionnaire. J Clin Epidemiol.

[B11] Washburn RA, Smith KW, Goldfield SR, McKinlay JB (1991). Reliability and physiologic correlates of the Harvard Alumni Activity Survey in a general population. J Clin Epidemiol.

[B12] Cox DR, Oakes D (1984). Analysis of survival data.

[B13] Tehard B, Friedenreich CM, Oppert JM, Clavel-Chapelon F (2006). Effect of physical activity on women at increased risk of breast cancer: results from the E3N cohort study. Cancer Epidemiol Biomarkers Prev.

[B14] Peplonska B, Lissowska J, Hartman TJ, Szeszenia-Dabrowska N, Blair A, Zatonski W, Sherman ME, Garcia-Closas M, Brinton LA (2008). Adulthood lifetime physical activity and breast cancer. Epidemiology.

[B15] Dallal CM, Sullivan-Halley J, Ross RK, Wang Y, Deapen D, Horn-Ross PL, Reynolds P, Stram DO, Clarke CA, Anton-Culver H, Ziogas A, Peel D, West DW, Wright W, Bernstein L (2007). Long-term recreational physical activity and risk of invasive and in situ breast cancer: the California teachers study. Arch Intern Med.

[B16] Slattery ML, Edwards S, Murtaugh MA, Sweeney C, Herrick J, Byers T, Giuliano AR, Baumgartner KB (2007). Physical activity and breast cancer risk among women in the southwestern United States. Ann Epidemiol.

[B17] McTiernan A, Kooperberg C, White E, Wilcox S, Coates R, Adams-Campbell LL, Woods N, Ockene J, Women's Health Initiative Cohort Study (2003). Recreational physical activity and the risk of breast cancer in postmenopausal women: the Women's Health Initiative Cohort Study. JAMA.

[B18] Lagerros YT, Hsieh SF, Hsieh CC (2004). Physical activity in adolescence and young adulthood and breast cancer risk: a quantitative review. Eur J Cancer Prev.

[B19] John EM, Horn-Ross PL, Koo J (2003). Lifetime physical activity and breast cancer risk in a multiethnic population: the San Francisco Bay area breast cancer study. Cancer Epidemiol Biomarkers Prev.

[B20] Lee IM, Cook NR, Rexrode KM, Buring JE (2001). Lifetime physical activity and risk of breast cancer. Br J Cancer.

[B21] Rockhill B, Willett WC, Hunter DJ, Manson JE, Hankinson SE, Colditz GA (1999). A prospective study of recreational physical activity and breast cancer risk. Arch Intern Med.

[B22] McTiernan A, Ulrich C, Slate S, Potter J (1998). Physical activity and cancer etiology: associations and mechanisms. Cancer Causes Control.

[B23] McTiernan A, Tworoger SS, Rajan KB, Yasui Y, Sorenson B, Ulrich CM, Chubak J, Stanczyk FZ, Bowen D, Irwin ML, Rudolph RE, Potter JD, Schwartz RS (2004). Effect of exercise on serum androgens in postmenopausal women: a 12-month randomized clinical trial. Cancer Epidemiol Biomarkers Prev.

[B24] McTiernan A, Tworoger SS, Ulrich CM, Yasui Y, Irwin ML, Rajan KB, Sorensen B, Rudolph RE, Bowen D, Stanczyk FZ, Potter JD, Schwartz RS (2004). Effect of exercise on serum estrogens in postmenopausal women: a 12-month randomized clinical trial. Cancer Res.

[B25] McTiernan A, Wu L, Chen C, Chlebowski R, Mossavar-Rahmani Y, Modugno F, Perri MG, Stanczyk FZ, Van Horn L, Wang CY, Women's Health Initiative Investigators (2006). Relation of BMI and physical activity to sex hormones in postmenopausal women. Obesity (Silver Spring).

[B26] Enger SM, Ross RK, Paganini-Hill A, Carpenter CL, Bernstein L (2000). Body size, physical activity, and breast cancer hormone receptor status: results from two case-control studies. Cancer Epidemiol Biomarkers Prev.

[B27] Thune I, Brenn T, Lund E, Gaard M (1997). Physical activity and the risk of breast cancer. N Engl J Med.

[B28] Shin A, Matthews CE, Shu XO, Gao YT, Lu W, Gu K, Zheng W (2008). Joint effects of body size, energy intake, and physical activity on breast cancer risk. Breast Cancer Res Treat.

[B29] Malin A, Matthews CE, Shu XO, Cai H, Dai Q, Jin F, Gao YT, Zheng W (2005). Energy balance and breast cancer risk. Cancer Epidemiol Biomarkers Prev.

[B30] Dirx MJ, Voorrips LE, Goldbohm RA, Brandt PA van den (2001). Baseline recreational physical activity, history of sports participation, and postmenopausal breast carcinoma risk in the Netherlands Cohort Study. Cancer.

[B31] Patel AV, Calle EE, Bernstein L, Wu AH, Thun MJ (2003). Recreational physical activity and risk of postmenopausal breast cancer in a large cohort of US women. Cancer Causes Control.

[B32] Moradi T, Adami HO, Ekbom A, Wedren S, Terry P, Floderus B, Lichtenstein P (2002). Physical activity and risk for breast cancer a prospective cohort study among Swedish twins. Int J Cancer.

[B33] Kruk J, Aboul-Enein HY (2003). Occupational physical activity and the risk of breast cancer. Cancer Detect Prev.

[B34] Coogan PF, Newcomb PA, Clapp RW, Trentham-Dietz A, Baron JA, Longnecker MP (1997). Physical activity in usual occupation and risk of breast cancer (United States). Cancer Causes Control.

[B35] Carpenter CL, Ross RK, Paganini-Hill A, Bernstein L (1999). Lifetime exercise activity and breast cancer risk among post-menopausal women. Br J Cancer.

[B36] Hirose K, Tajima K, Hamajima N, Inoue M, Takezaki T, Kuroishi T, Yoshida M, Tokudome S (1995). A large-scale, hospital-based case-control study of risk factors of breast cancer according to menopausal status. Jpn J Cancer Res.

[B37] Shoff SM, Newcomb PA, Trentham-Dietz A, Remington PL, Mittendorf R, Greenberg ER, Willett WC (2000). Early-life physical activity and postmenopausal breast cancer: effect of body size and weight change. Cancer Epidemiol Biomarkers Prev.

[B38] Colditz GA, Feskanich D, Chen WY, Hunter DJ, Willett WC (2003). Physical activity and risk of breast cancer in premenopausal women. Br J Cancer.

[B39] Sesso HD, Paffenbarger RS, Lee IM (1998). Physical activity and breast cancer risk in the College Alumni Health Study (United States). Cancer Causes Control.

[B40] Breslow RA, Ballard-Barbash R, Munoz K, Graubard BI (2001). Long-term recreational physical activity and breast cancer in the National Health and Nutrition Examination Survey I epidemiologic follow-up study. Cancer Epidemiol Biomarkers Prev.

[B41] Moore DB, Folsom AR, Mink PJ, Hong CP, Anderson KE, Kushi LH (2000). Physical activity and incidence of postmenopausal breast cancer. Epidemiology.

[B42] Yang D, Bernstein L, Wu AH (2003). Physical activity and breast cancer risk among Asian-American women in Los Angeles: a case-control study. Cancer.

[B43] McTiernan A, Stanford JL, Weiss NS, Daling JR, Voigt LF (1996). Occurrence of breast cancer in relation to recreational exercise in women age 50–64 years. Epidemiology.

[B44] Friedenreich CM, Bryant HE, Courneya KS (2001). Case-control study of lifetime physical activity and breast cancer risk. Am J Epidemiol.

[B45] D'Avanzo B, Nanni O, La Vecchia C, Franceschi S, Negri E, Giacosa A, Conti E, Montella M, Talamini R, Decarli A (1996). Physical activity and breast cancer risk. Cancer Epidemiol Biomarkers Prev.

[B46] Matthews CE, Shu XO, Jin F, Dai Q, Hebert JR, Ruan ZX, Gao YT, Zheng W (2001). Lifetime physical activity and breast cancer risk in the Shanghai Breast Cancer Study. Br J Cancer.

[B47] McTiernan A (2008). Mechanisms linking physical activity with cancer. Nat Rev Cancer.

[B48] Adams SA, Matthews CE, Hebert JR, Moore CG, Cunningham JE, Shu XO, Fulton J, Gao Y, Zheng W (2006). Association of physical activity with hormone receptor status: the Shanghai Breast Cancer Study. Cancer Epidemiol Biomarkers Prev.

[B49] Bernstein L, Patel AV, Ursin G, Sullivan-Halley J, Press MF, Deapen D, Berlin JA, Daling JR, McDonald JA, Norman SA, Malone KE, Strom BL, Liff J, Folger SG, Simon MS, Burkman RT, Marchbanks PA, Weiss LK, Spirtas R (2005). Lifetime recreational exercise activity and breast cancer risk among black women and white women. J Natl Cancer Inst.

[B50] Bardia A, Hartmann LC, Vachon CM, Vierkant RA, Wang AH, Olson JE, Sellers TA, Cerhan JR (2006). Recreational physical activity and risk of postmenopausal breast cancer based on hormone receptor status. Arch Intern Med.

[B51] Britton JA, Gammon MD, Kelsey JL, Brogan DJ, Coates RJ, Schoenberg JB, Potischman N, Swanson CA, Stanford JL, Brinton LA (2000). Characteristics associated with recent recreational exercise among women 20 to 44 years of age. Women Health.

[B52] Sallis JF, Saelens BE (2000). Assessment of physical activity by self-report: status, limitations, and future directions. Res Q Exerc Sport.

[B53] Leitzmann MF, Rimm EB, Willett WC, Spiegelman D, Grodstein F, Stampfer MJ, Colditz GA, Giovannucci E (1999). Recreational physical activity and the risk of cholecystectomy in women. N Engl J Med.

